# The updated 2023 staging of endometrial cancer: tips for MRI interpretation

**DOI:** 10.1007/s00261-025-05058-9

**Published:** 2025-06-13

**Authors:** Diogo Miguel Machado Pereira, Alfonso Iglesias Castañon, Mercedes Arias Gonzalez, Alfonso Escobar Villalba, Marlon Francisco Ferreira Polli, Marta Herreros Villaraviz, Jorge Mañas Uxo, Beatriz Nieto Baltar, Angel Nieto Parga

**Affiliations:** Diagnostic Imaging Unit, Galaria EPSS, Vigo, Spain

**Keywords:** Endometrial cancer, FIGO 2023 staging, MRI, Histopathology, Molecular classification

## Abstract

**Purpose:**

This study aims to highlight the major modifications introduced in the FIGO 2023 staging system for endometrial cancer (EC) and their implications for MRI interpretation.

**Methods:**

This pictorial essay was based on a retrospective review of 27 histologically confirmed cases of endometrial cancer (EC) imaged between 2009 and 2023 at our institution. Cases were selected to represent a broad spectrum of FIGO 2009 and FIGO 2023 stages, emphasizing features with updated staging implications. Inclusion criteria were availability of preoperative pelvic MRI and complete histopathological data, including molecular classification when available. Exclusion criteria included suboptimal image quality or incomplete clinical records. MRI assessments were performed by two radiologists with 25 years of experience in gynecological imaging, respectively. In illustrative examples where inter-reader differences arose, consensus was reached after joint review; however, no formal inter-reader agreement statistics (e.g., kappa values) were calculated, given the descriptive nature of the study.

**Results:**

The revised FIGO 2023 staging incorporates molecular subtypes, refines classification criteria, and improves the prognostic significance of MRI findings in EC staging.

**Conclusion:**

The integration of histopathology, molecular markers, and MRI features enhances diagnostic accuracy and treatment planning.

## Introduction

The new FIGO 2023 staging [1] for endometrial cancer (EC) includes various histological types, tumor patterns, and molecular classification [2], as they affect prognosis and enable a more appropriate treatment planning [3].

New histopathological variables included in the 2023 FIGO staging for EC:

- Two histological types [4]:
  - Non-aggressive tumors: low-grade endometrioid carcinoma (grades G1 and G2).
  - Aggressive tumors: high-grade endometrioid carcinoma (grade G3) and non-endometrioid carcinoma (including serous, clear cell, undifferentiated, mixed, mesonephric-like, gastrointestinal-type mucinous carcinomas, and carcinosarcomas).
- Incorporation of molecular subtypes [5]:
  - POLE mutation (associated with favorable prognosis).
  - p53 mutation (associated with poor prognosis).
    The integration of molecular classification (POLE, p53, MMRd, NSMP) in the 2023 FIGO staging system reflects prognostic categories established through tissue-based analysis. MRI findings alone are not sufficient to determine these subtypes; however, certain imaging features—such as large heterogeneous masses, deep myometrial invasion, or lymph node involvement—may raise suspicion for aggressive behavior, particularly in p53-abnormal tumors. In clinical practice, molecular data are increasingly available preoperatively through endometrial biopsy and are used in conjunction with MRI findings to guide staging and management decisions.
- Lymphovascular space invasion (LVSI) [6]:
  Substantial LVSI is defined as involvement of five or more vessels, according to recent guidelines and pathological consensus [6]. This threshold holds prognostic value, as substantial LVSI is a well-established risk factor for metastases and recurrence in endometrial carcinoma, particularly in non-aggressive histological types and in early-stage endometrial carcinoma. Conversely, no LVSI or focal LVSI (less than 5 vessels involved) have been associated with significantly better prognostic outcomes. In the FIGO 2023 system, the presence of substantial LVSI may lead to upstaging to Stage IIB, even in tumors with otherwise favorable features.
  While MRI cannot directly assess LVSI, certain imaging features may raise suspicion, such as irregular or spiculated tumor margins, disruption of the junctional zone, or ill-defined tumor-myometrium interfaces. Nevertheless, more studies should be done in order to define all the radiological spectrum of the substantial or focal linfovascular invasion. Awareness of histological LVSI status is crucial when interpreting MRI for staging and treatment planning.
- This review summarizes the updates in the FIGO 2023 staging, emphasizing the differences compared to the FIGO 2009 system. It also correlates MRI findings with the updated staging and highlights imaging data crucial for managing patients with EC [7, 8].

### Changes between the 2009 and 2023 FIGO staging systems for EC (Table 1).

- Stage IA subdivided into:
  - IA1: no myometrial invasion (non-aggressive EC).
  - IA2: myometrial invasion < 50% (non-aggressive EC).
  - IA3: ovarian involvement and myometrial invasion < 50% (non-aggressive EC).
- Stage IC: aggressive histological types limited to an endometrial polyp or confined to the endometrium.
- Stage II subdivided into:
  - IIA: cervical stromal invasion (non-aggressive EC).
  - IIB: myometrial invasion (any EC aggressiveness).
- Stage IIIA subdivided into:
  - IIIA1: adnexal invasion.
  - IIIA2: uterine serosal invasion.
- Stage IIIB subdivided into:
  - IIIB1: vaginal and/or parametrial invasion.
  - IIIB2: pelvic peritoneal invasion.
- Stage IIIC subdivided into:
  - IIIC1: pelvic lymph nodes.
  - IIIC2: infrarenal para-aortic lymph nodes.
  - Each with micrometastasis (i) or macrometastasis (ii).
- Stage IVB subdivided into:
  - IVB: peritoneal metastases beyond the pelvis.
  - IVC: distant metastases, including inguinal and intra-abdominal lymph nodes above the renal vessels.

**Table 1:** Changes in FIGO staging for endometrial cancer (EC)

FIGO 2009 stage	FIGO 2023 stage
IA	IA1, IA2, IA3
IB	IB, IC
II	IIA, IIB, IIC
IIIA	IIIA1, IIIA2
IIIB	IIIB1, IIIB2
IIIC1	IIIC1i, IIIC1ii
IIIC2	IIIC2i, IIIC2ii
IVB	IVB, IVC

*IA3*: Non-aggressive histology, with ≥ 50% myometrial invasion and no LVSI; *IC*: Aggressive histology with invasion < 50% and no LVSI; *IIA–IIC*: Reflect combined cervical stromal invasion and LVSI/molecular features; *IIIB1/IIIB2*: Denote vaginal vs. parametrial involvement; *IIIC1i/ii & IIIC2i/ii*: Indicate pelvic vs. para-aortic lymph node involvement with subclassification into isolated tumor cells/micrometastases (i) and macrometastases (ii); VB: Peritoneal metastasis beyond the pelvis; IVC: Distant organ or nodal metastases (e.g., lungs, suprarenal nodes)

**Implications of the 2023 FIGO Staging of EC in MRI: MRI plays a key role in identifying prognostic factors preoperatively**:

- Stage I Myometrial invasion.
- Stage II Cervical stroma invasion.
- Stage III: ovarian/fallopian involvement, uterine subserosal involvement, vaginal/parametrial invasion, peritoneal spread, pelvic/para-aortic lymph node metastasis.
- Stage IV: bladder or intestinal mucosal invasion, distant metastasis.

### 2023 FIGO stage I

Combination of histologic findings and presence and depth of myometrial invasion. Includes tumors confined to the uterine corpus; without LVSI; with a non-aggressive histological type (with or without myometrial invasion), or any aggressive histological type confined to the endometrium (no myometrial invasion).

- **IA1** Non-aggressive, no LVSI, in an endometrial polyp or confined to the endometrium.
- **IA2** Non-aggressive, no LVSI, myometrial invasion < 50%.
- **IA3** Non-aggressive, no LVSI, myometrial invasion < 50%, ovary involvement.
- **IB** Non-aggressive, no LVSI, myometrial invasion ≥ 50%.
- **IC** Aggressive histological types limited to an endometrial polyp or confined to the endometrium.

**Fig. 1:**
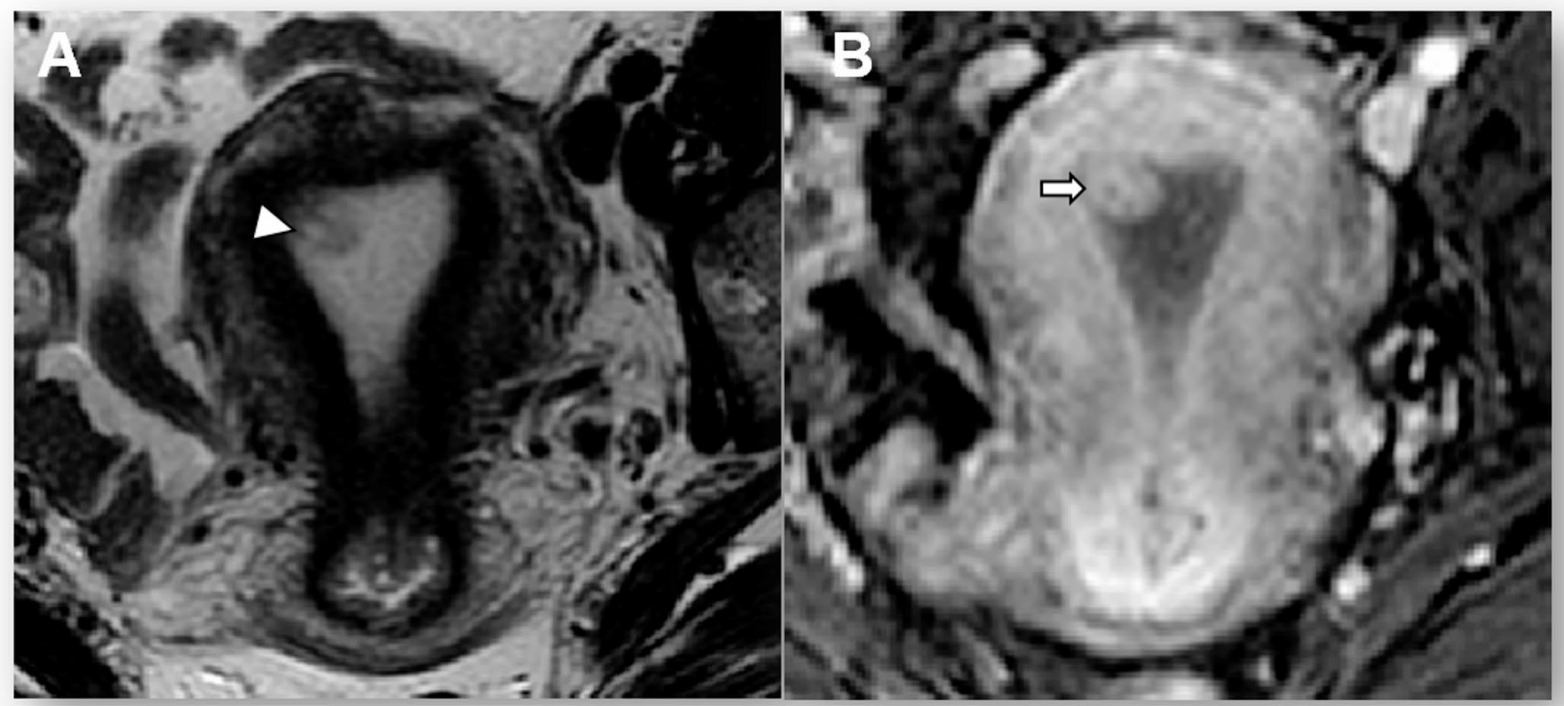
Stage IA1 grade 1 endometrioid carcinoma, no LVSI, in a 48-year-old woman. **A** Axial oblique T2-weighted fast spin-echo (FSE) image (TR/TE: 4500/100 ms, slice thickness 3 mm) and **B** contrast-enhanced 3D T1-weighted fat-suppressed LAVA sequence (TR/TE: 4.2/2.1 ms; slice thickness: 2 mm; post-gadolinium, delayed phase at 4 min) show an endometrial polyp without myometrial invasion (arrow). On the T2-WI, hyperintense cystic spaces are seen within the polyp (arrowhead)

In the revised FIGO 2023 cancer staging, histologically non-aggressive tumors limited to an endometrial polyp are classified as Stage IA1 (previous FIGO staging was Stage IA) (Fig. 1). It is important to confirm/exclude myometrial invasion, as it is an essential prognostic risk factor [9, 10].

**Fig. 2:**
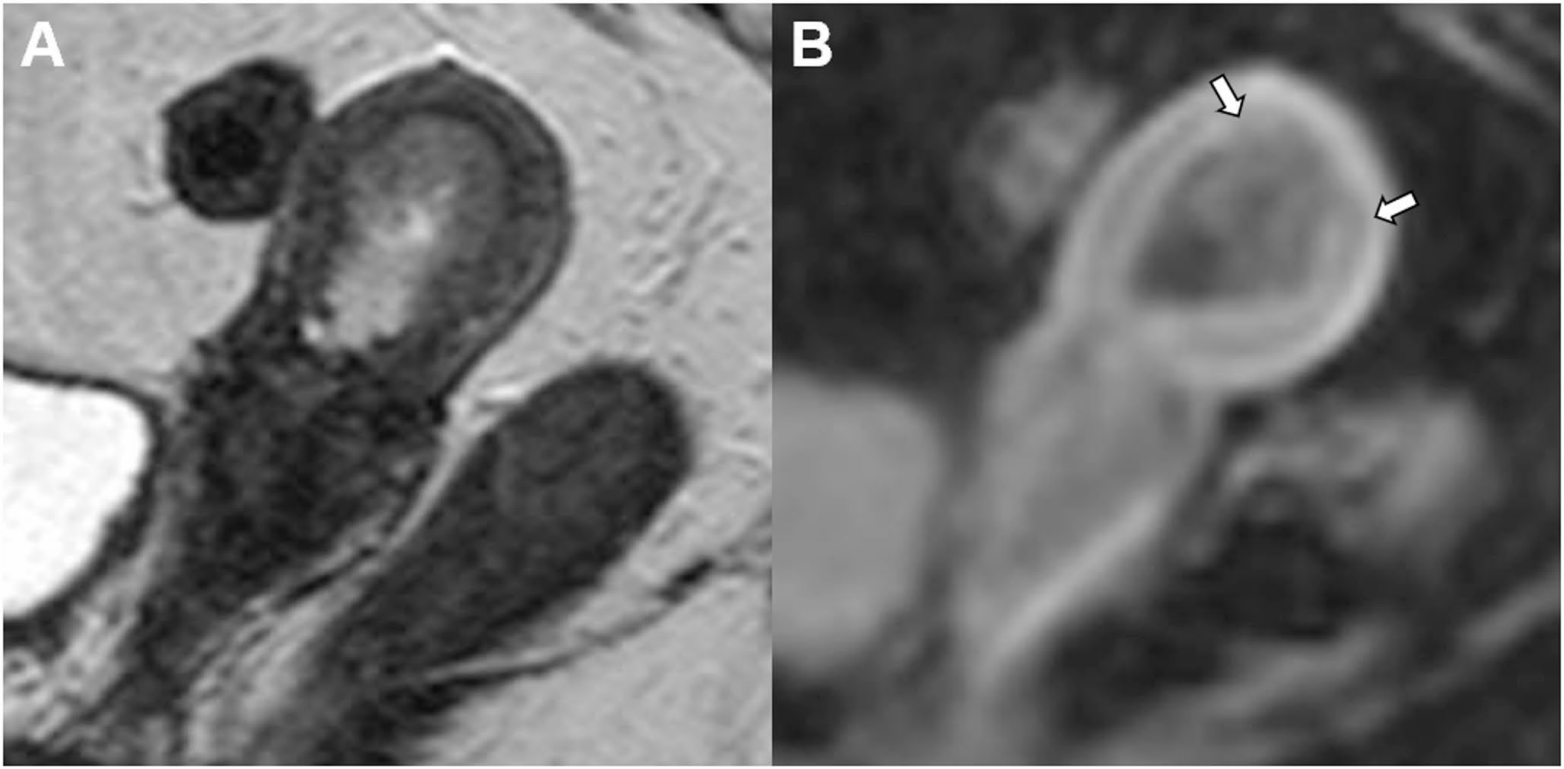
Stage IA2 grade 2 endometrioid carcinoma, no LVSI, and myometrial invasion < 50% in a 58-year-old woman. **A** Sagittal T2-weighted fast spin-echo (FSE) image (TR/TE: 4500/100 ms; slice thickness: 3 mm) and **B** contrast-enhanced 3D T1-weighted fat-suppressed LAVA sequence (TR/TE: 4.2/2.1 ms; slice thickness: 2 mm; post-gadolinium, delayed phase at 4 min) show an endometrial mass with superficial myometrial invasion (arrows)

Low-grade endometrial carcinoma extending < 50% into the myometrium are classified as Stage IA2 (previously Stage IA)(Fig. 2). Disruption of the junctional zone in uterine fundus is better appreciated on contrast-enhanced T1-weighted fat-saturated MR images [11].

**Fig. 3:**
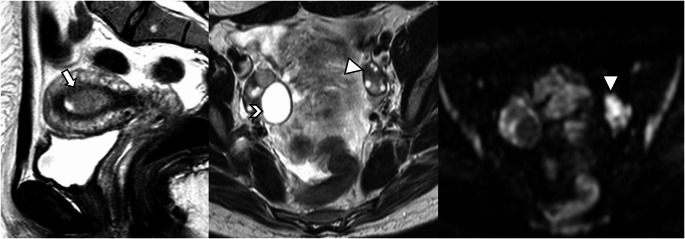
Stage IA3 grade 2 endometrioid carcinoma, no LVSI, confined to the endometrium and left ovary in a 60-year-old woman. **A** Sagittal T2-weighted fast spin-echo (FSE) image (TR/TE: 4500/100 ms; slice thickness: 3 mm) shows a mass confined to the endometrium without myometrial invasion (arrow). **B** Axial T2-weighted fast spin-echo (FSE) image (TR/TE: 4500/100 ms; slice thickness: 3 mm) and (**C** diffusion-weighted image (DWI) with b = 800 s/mm^2^ show a nodule in the left ovary without capsule invasion (arroweads). Cysts in the right ovary (chevron). Pathology confirmed low grade endometrial carcinoma involving both endometrium and left ovary

Low-grade endometrial carcinoma involving both endometrium and ovary, and meeting specific criteria are classified as Stage IA3 (previously Stage IIIA) (Fig. 3). These must be distinguished from extensive EC spread to the ovary (Stage IIIA1) by the following criteria:

1. No more than superficial myometrial invasion (< 50%).
2. Absence of LVSI.
3. Absence of additional metastases.
4. Unilateral ovarian tumor, confined to the ovary, without capsule invasion/rupture (pT1a).

Patients with low-grade endometrioid carcinomas limited to the uterus and the ovaries are considered to have a good prognosis and no adjuvant treatment is recommended [3].

**Fig. 4:**
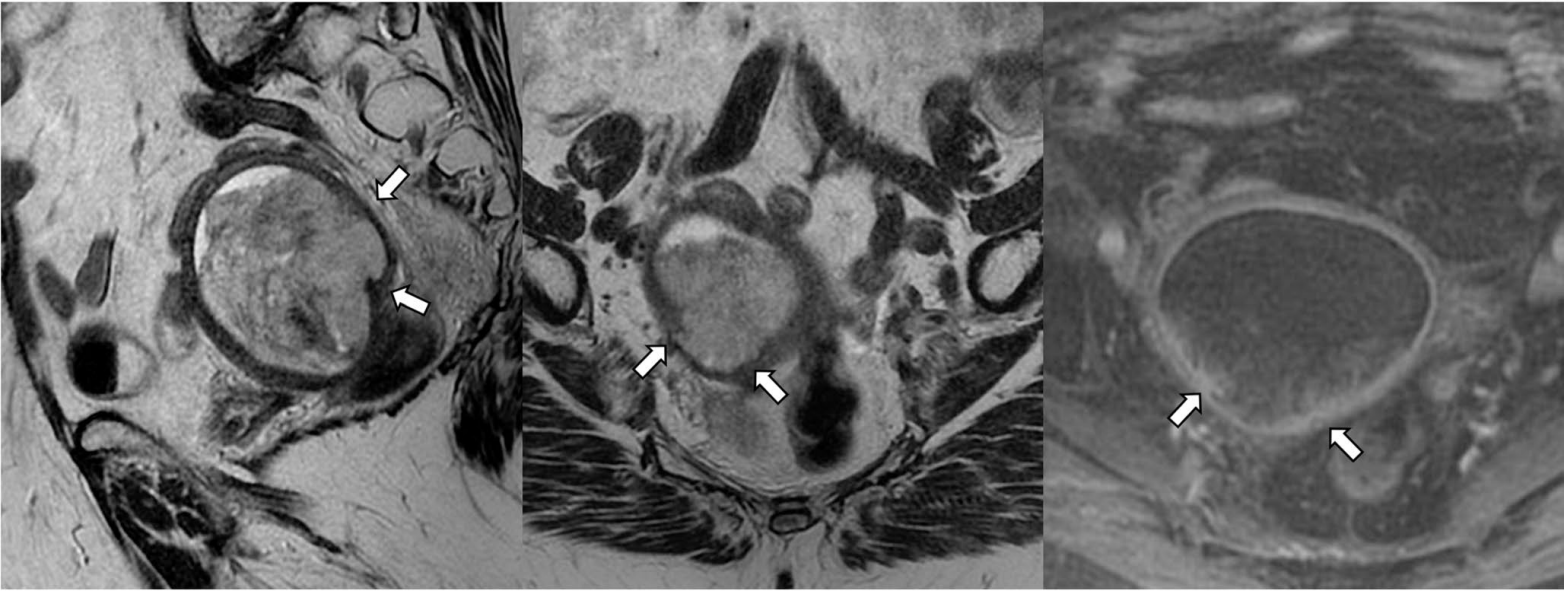
Stage IB grade 2 endometrioid carcinoma, no LVSI in a 60-year-old woman. **A** Sagittal T2-weighted fast spin-echo (FSE) image (TR/TE: 4500/100 ms; slice thickness: 3 mm) and **B** axial oblique T2-weighted fast spin-echo (FSE) image (TR/TE: 4500/100 ms; slice thickness: 3 mm) show an endometrial mass with myometrial invasion (arrows). **C** Axial contrast-enhanced 3D T1-weighted fat-suppressed LAVA sequence (TR/TE: 4.2/2.1 ms; slice thickness: 2 mm; post-gadolinium, delayed phase at 4 min) shows disruption of the junctional zone. Pathology confirmed low grade endometrioid carcinoma involving > 50% of the myometrium

Low-grade endometrial carcinomas which extend more than 50% into the myometrium are classified as Stage IB (unchanged from previous staging) (Fig. 4). When the tumor exceeds 50% into the myometrium, the presence of an intact stripe of normal outer myometrium excludes uterine serosa invasion [12, 13].

**Fig. 5:**
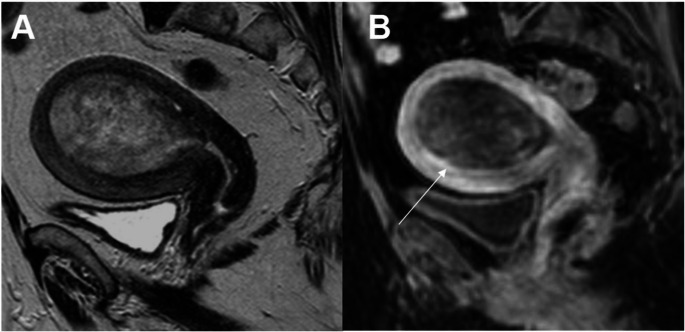
Stage IC carcinosarcoma, no LVSI in a 67-year-old woman. **A** Sagittal T2-weighted fast spin-echo (FSE) image (TR/TE: 4500/100 ms; slice thickness: 3 mm) and **B** Sagittal contrast-enhanced 3D T1-weighted fat-suppressed LAVA sequence (TR/TE: 4.2/2.1 ms; slice thickness: 2 mm; post-gadolinium, delayed phase at 3 min) show a bulky endometrial tumor distending the endometrial cavity without myometrial invasion. Preservation of the junctional zone is better appreciated on dynamic images after contrast (arrow). Pathology confirmed carcinosarcoma limited to the endometrium

Any aggressive histological type without myometrial involvement is Stage IC (previously Stage IA) (Fig. 5). The risk of extrauterine disease in aggressive endometrial carcinomas does not correlate with the depth of myometrial invasion as nodal or intraperitoneal metastases can occur even when there is no myometrial invasion [14].

#### Tips for MRI interpretation: stage I

- It is important to confirm or exclude myometrial invasion, as it is a key prognostic risk factor.
- The depth of myometrial invasion is best measured on axial oblique images perpendicular to the endometrial cavity.
- The extent of myometrial infiltration by carcinoma is categorized as: none; <50%; or ≥ 50%.
- Dynamic multiphase contrast-enhanced imaging (DCE) improves the accuracy of assessing myometrial invasion by demonstrating a smooth, uninterrupted band of subendometrial enhancement and helping to exclude superficial myometrial invasion.

### 2023 FIGO stage II

Combination of histological findings, myometrial invasion and cervical stroma invasion.

Includes cervical stroma invasion in non-aggressive histological types without extrauterine extension, non-aggressive histological types with substantial LVSI, or aggressive histological types with myometrial invasion.

- **IIA** Cervical stroma invasion in non-aggressive tumors with no LVSI.
- **IIB** Substantial LVSI in non-aggressive histological types.
- **IIC** Aggressive histological type with any myometrial invasion.

**Fig. 6:**
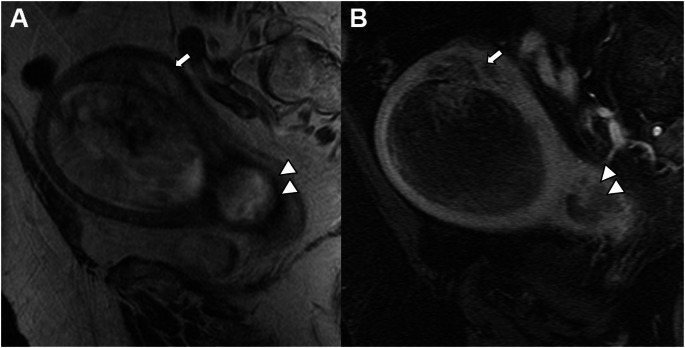
Stage IIA grade 2 endometrioid carcinoma, no LVSI, in a 65-year-old woman. **A** Sagittal T2-weighted fast spin-echo (FSE) image (TR/TE: 4500/100 ms; slice thickness: 3 mm) and **B** sagittal contrast-enhanced 3D T1-weighted fat-suppressed LAVA sequence (TR/TE: 4.2/2.1 ms; slice thickness: 2 mm; post-gadolinium, delayed phase at 4 min) show disruption of the junctional zone (arrow). The tumor extends more than 50% into the myometrium and into the cervical canal with disruption of the cervical stroma (arrowheads), best seen on delayed phase images

In the revised FIGO 2023 endometrial cancer staging, non-aggressive histological tumors without extrauterine extension are classified as Stage IIA (previously Stage II) (Fig. 6). Disruption of the cervical stroma is better appreciated on delayed contrast-enhanced T1-weighted fat-saturated MR images [12, 15].

**Fig. 7:**
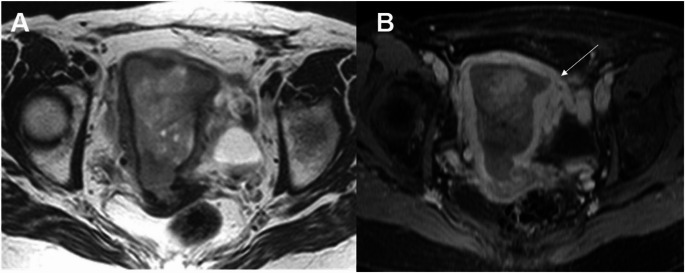
Stage IIC carcinosarcoma, no LVSI in a 67-year-old woman. **A** Axial oblique T2-weighted fast spin-echo (FSE) image (TR/TE: 4500/100 ms; slice thickness: 3 mm) and **B** a contrast-enhanced 3D T1-weighted fat-suppressed LAVA sequence (TR/TE: 4.2/2.1 ms; slice thickness: 2 mm; post-gadolinium, delayed phase at 4 min) show a mass invading the superficial myometrium and a peripheral rim of normally enhancing myometrium (arrow)

Aggressive histological tumors with any myometrial involvement are classified as Stage IIC (previously Stage IB) (Fig. 7). This change reflects evidence from randomized trials, prospective cohort studies, large database series, and retrospective reports that consistently show aggressive histological types have a markedly higher recurrence rate [16, 17].

#### Tips for MRI interpretation: stage II

- Assessment of cervical stroma invasion best depicted on delayed images after intravenous gadolinium administration.
- Suspect cervical stroma invasion when the cervical mucosa enhancement is disrupted by a poorly enhancing tumor on delayed images (4–5 min after contrast).
- Continuous enhancement of the cervical mucosa on delayed phase images excludes cervical stroma invasion.
- Distinguish between tumor protruding from the endometrial cavity into the endocervix (stage I), and cervical stroma invasion (stage II).

### 2023 FIGO stage III

Includes local and/or regional spread of the tumor of any histological type.

- **IIIA**: Direct or metastatic invasion of uterine serosa, adnexa, or both.
  - **IIIA1**: Spread to ovary or fallopian tube (except for IA3 cases).
  - **IIIA2**: Invasion of uterine serosa or subserosa.
- **IIIB**: Direct or metastatic invasion of the vagina and/or parametria or pelvic peritoneum.
  - **IIIB1**: Metastasis or direct invasion of the vagina and/or parametria.
  - **IIIB2**: Metastasis to the pelvic peritoneum.
- **IIIC**: Metastasis to pelvic or para-aortic lymph nodes or both.
  - **IIIC1**: Metastasis to pelvic lymph nodes.
    - **IIIC1i**: Micrometastasis.
    - **IIIC1ii**: Macrometastasis.
  - **IIIC2**: Metastasis in infrarenal para-aortic lymph nodes with or without pelvic lymph node metastasis.
    - **IIIC2i**: Micrometastasis.
    - **IIIC2ii**: Macrometastasis.

**Fig. 8:**
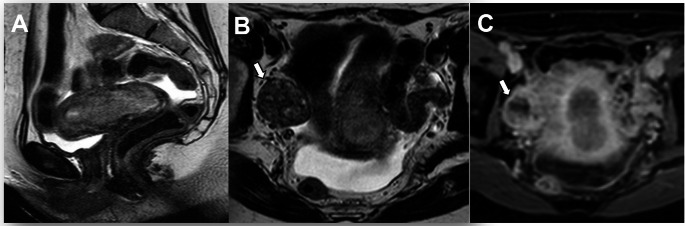
Stage IIIA1 grade 3 endometrioid carcinoma, in a 70-year-old woman. **A** Sagittal and **B** axial T2-weighted fast spin-echo (FSE) image (TR/TE: 4500/100 ms; slice thickness: 3 mm) and **C**) axial contrast-enhanced 3D T1-weighted fat-suppressed LAVA sequence (TR/TE: 4.2/2.1 ms; slice thickness: 2 mm; post-gadolinium, delayed phase at 4 min) show a large endometrial mass distending the uterine cavity with myometrial invasion, extending into the cervical channel with stromal invasion. On axial T2WI and on dynamic contrast-enhanced images a right parauterine heterogeneous mass is demonstrated (arrows). Right ovarian metastasis was confirmed by pathology

In the revised FIGO 2023 endometrial cancer staging, any histological subtype with ovarian metastasis is classified as Stage IIIA1 (previously Stage IIIA) (Fig. 8). Spread of the EC to the ovary (Stage IIIA1) must be distinguished from low-grade EC involving < 50% myometrium and ovaries (Stage IA3). Patients with extensive spread of the EC to the ovary (Stage IIIA1) have a worse prognosis, and adjuvant treatment is recommended [18–20].

**Fig. 9:**
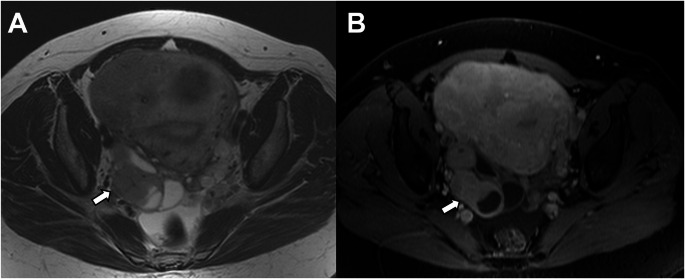
Stage IIIA1 carcinosarcoma in a 72-year-old woman. **A** T2-weighted fast spin-echo (FSE) image (TR/TE: 4500/100 ms; slice thickness: 3 mm) and **B** axial contrast-enhanced 3D T1-weighted fat-suppressed LAVA sequence (TR/TE: 4.2/2.1 ms; slice thickness: 2 mm; post-gadolinium, delayed phase at 4 min) show absent myometrial signal intensity and a right adnexal mass (arrow). Pathology confirmed involvement of the myometrium and right adnexa by carcinosarcoma

Any histological subtype with direct adnexal extension is also classified as Stage IIIA1 (previously Stage IIIA) (Fig. 9). Subclassifications of Stage III were defined to better reflect tumor behavior, especially in high-grade and non-endometrioid carcinomas [21].

**Fig. 10:**
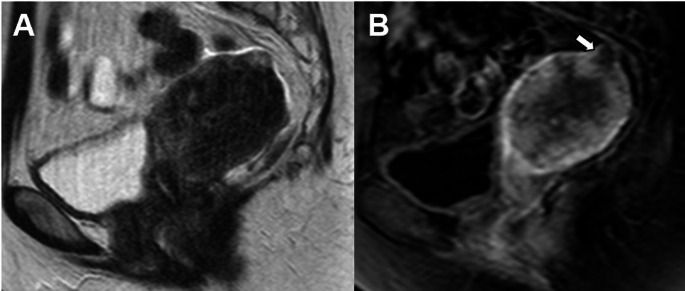
Stage IIIA2 serous carcinoma in a 70-year-old woman. **A** Sagittal T2-weighted fast spin-echo (FSE) image (TR/TE: 4500/100 ms; slice thickness: 3 mm) and **B** sagittal contrast-enhanced 3D T1-weighted fat-suppressed LAVA sequence (TR/TE: 4.2/2.1 ms; slice thickness: 2 mm; post-gadolinium, delayed phase at 4 min) show absent normal myometrial signal intensity and an irregular uterine contour at the fundus (arrows). Pathology confirmed serous carcinoma with involvement of the uterine serosa

Uterine serosa infiltration is classified as Stage IIIA2 (previously Stage IIIA) (Fig. 10). The subclassification of Stage III aims to better reflect prognosis and support more appropriate treatment decisions [1].

**Fig. 11:**
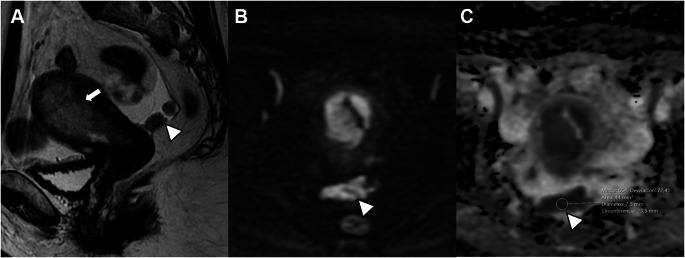
Stage IIIB2 uterine serous carcinoma in a 70-year-old woman. **A** Sagittal T2-weighted fast spin-echo (FSE) image (TR/TE: 4500/100 ms; slice thickness: 3 mm), **B** axial diffusion-weighted image (DWI) with b = 1000 s/mm^2^, and **C** ADC map show a uterine mass (arrow) and peritoneal implants in the Douglas pouch (arrowheads). ADC value in the peritoneal implant is 0.65 × 10^−3^ mm^2^/s

Pelvic peritoneal metastases are now classified as Stage IIIB2, distinct from peritoneal involvement that extends beyond the pelvis, which is classified as Stage IVB (Fig. 11). Treatment decisions vary significantly depending on whether peritoneal carcinomatosis is limited to the pelvis (Stage IIIB2) or extends beyond (Stage IVB). The anatomical landmark of the pelvis is the line between the anterior superior iliac spines [22].

**Fig. 12:**
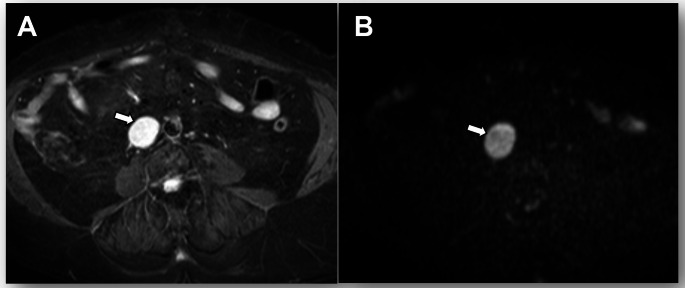
Stage IIIC2 grade 2 endometrioid carcinoma in a 63-year-old woman. **A** Axial fat-suppressed T2-weighted fast spin-echo (FSE) image (TR/TE: 4500/100 ms; slice thickness: 3 mm) shows an enlarged para-aortic lymph node (arrow). **B** Axial diffusion-weighted image (DWI) with b = 800 s/mm^2^ shows a hyperintense lymph node (arrow). ADC value in the node is 0.94 × 10^−3^ mm^2^/s. Pathological analysis confirmed lymph node metastases

Metastasis to para-aortic lymph nodes up to the renal vessels is classified as Stage IIIC2, distinct from intra-abdominal or para-aortic lymph nodes above the renal vessels, which are classified as Stage IVC (Fig. 12). Staging distinguishes para-aortic lymph nodes as Stage III vs. Stage IV using the renal vessels as the boundary. Lymph node metastases alter prognosis and modify the therapeutic approach [23].

## Tips for MRI interpretation: stage III

- Local or regional tumor spread is defined as “beyond the uterus but not outside the true pelvis.”
- The anatomical landmark of the pelvis is the line connecting the anterior superior iliac spines.
- MRI features suspicious for lymph node involvement include: size > 1 cm, multiplicity, irregular contour, necrosis, and signal intensity similar to the primary tumor.
- Lymph node and peritoneal carcinomatosis detection improves with DWI (nodes appear very bright), though correlation with T2-WI is always advised.

### 2023 FIGO stage IV

Includes bladder and/or intestinal mucosa invasion and/or distant metastasis.

- **IVA**: Bladder and/or intestinal mucosa invasion.
- **IVB**: Abdominal peritoneal metastasis beyond the pelvis.
- **IVC**: Distant metastasis (lungs, liver, brain, or bone), including metastasis to any extra- or intra-abdominal lymph nodes above the renal vessels.

**Fig. 13:**
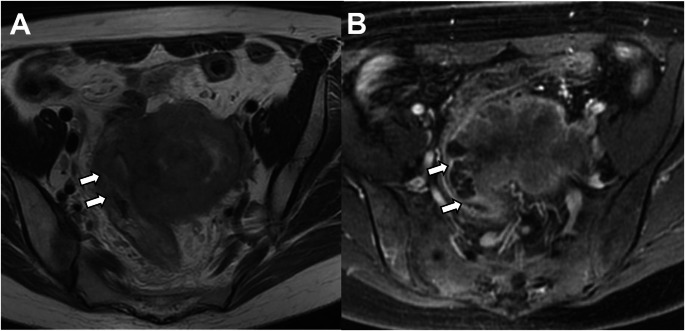
Stage IVA grade 3 endometrioid adenocarcinoma in a 77-year-old woman. **A** Axial T2-weighted fast spin-echo (FSE) image (TR/TE: 4500/100 ms; slice thickness: 3 mm) and **B** axial contrast-enhanced 3D T1-weighted fat-suppressed LAVA sequence (TR/TE: 4.2/2.1 ms; slice thickness: 2 mm; post-gadolinium, delayed phase at 4 min) show a large endometrial mass with myometrial invasion, extending through the myometrium and serosal surface directly into the rectal mucosa (arrows). Best seen on delayed contrast-enhanced image

In the revised FIGO 2023 endometrial cancer staging, spread to the intestinal mucosa is classified as Stage IVA (Fig. 13). There is no difference compared to the previous 2009 FIGO staging system. Intestinal and/or bladder mucosal involvement must be confirmed endoscopically [24].

**Fig. 14:**
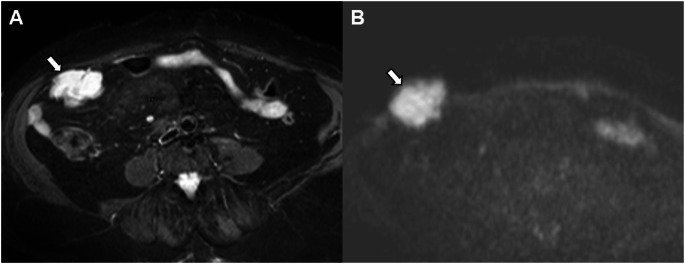
Extended MR study of the abdomen in a 65-year-old woman with uterine serous carcinoma. **A** Axial fat-suppressed T2-weighted fast spin-echo (FSE) image (TR/TE: 4500/100 ms; slice thickness: 3 mm) and **B** diffusion-weighted image (DWI) with b = 1000 s/mm^2^ show a peritoneal implant beyond the pelvis (arrow). Pathological analysis confirmed metastasis

Extrapelvic peritoneal metastases are now classified as Stage IVB, distinct from those with peritoneal involvement confined to the pelvis, which are classified as Stage IIIB2 (Fig. 14). This change was introduced to better align staging with clinical decision-making, particularly surgical indications, which vary significantly between pelvic-limited and extrapelvic peritoneal carcinomatosis [25].

#### Tips for MRI interpretation: stage IV

- Extend MRI protocols to include the upper abdomen to evaluate nodal metastases and the presence of peritoneal carcinomatosis for accurate staging.
- Preserved fat planes between the tumor and bladder or rectum exclude Stage IVA with high accuracy, potentially avoiding the need for cystoscopy or rectosigmoidoscopy.
- Peritoneal metastases beyond the pelvis indicate Stage IVB.
- Distant metastases, and/or involvement of inguinal or suprarenal lymph nodes, indicate Stage IVC.

### Changes in the 2023 FIGO staging of EC based on location and histological type

Tumors confined to the endometrium.

– Stage IA1 for non-aggressive tumors.
– Stage IC for aggressive tumors.

Tumors that penetrate the inner myometrium.

– Stage IA2 for non-aggressive tumors.
– Stage IIC for aggressive tumors.

Tumors with uterine subserosa involvement.

- Stage IIIA2.

Tumors with peritoneal involvement.

– Stage IIIB2 for isolated pelvic peritoneum involvement.
– Stage IVB for peritoneal involvement beyond the pelvis.

Nodal metastases.

– Stage IIIC1i/IIIC2i = micrometastases (≤ 2 mm).
– Stage IIIC1ii/IIIC2ii = macrometastases (> 2 mm).

Why is MRI useful in 2023 FIGO Staging System for EC?

MRI plays a crucial role in the preoperative assessment of key prognostic factors such as tumor size; depth of myometrial invasion (none; < 50%; or ≥ 50%); cervical stroma invasion; extrauterine spread (including ovarian and/or fallopian tube involvement, uterine subserosal extension, vaginal and/or parametrial invasion, peritoneal carcinomatosis, bladder or bowel mucosal involvement); and lymph node size.

Incorporating these imaging findings into the staging process enables more personalized and effective treatment planning for each patient.

## Conclusions

The updated 2023 FIGO endometrial cancer staging system represents a major advancement toward more precise and individualized staging by integrating anatomical, pathological, and molecular parameters. The inclusion of pathological and molecular variables enhances prognostic accuracy and guides therapeutic decision-making.

MRI plays a vital role in the preoperative staging of endometrial cancer by accurately identifying critical prognostic factors, thereby improving treatment planning. Consequently, it is essential for radiologists to be familiar with relevant histopathological data before assigning a prognostic stage.

## Data Availability

No datasets were generated or analysed during the current study.
